# A single-nucleotide-polymorphism-based genotyping assay for simultaneous detection of different carbendazim-resistant genotypes in the *Fusarium graminearum* species complex

**DOI:** 10.7717/peerj.2609

**Published:** 2016-10-25

**Authors:** Hao Zhang, Balázs Brankovics, Theo A.J. van der Lee, Cees Waalwijk, Anne A.D. van Diepeningen, Jin Xu, Jingsheng Xu, Wanquan Chen, Jie Feng

**Affiliations:** 1 Institute of Plant Protection, Chinese Academy of Agricultural Sciences, Beijing, China; 2 CBS-KNAW Fungal Biodiversity Centre, Royal Netherlands Academy of Arts and Sciences, Utrecht, Netherlands; 3 Institute for Biodiversity and Ecosystem Dynamics, University of Amsterdam, Amsterdam, Netherlands; 4 Department of Biointeractions and Plant Health, Wageningen University and Research, Wageningen, Netherlands

**Keywords:** *Fusarium graminearum* species complex, Luminex, MBC resistance

## Abstract

The occurrence resistance to methyl benzimidazole carbamates (MBC)-fungicides in the *Fusarium graminearum* species complex (FGSC) is becoming a serious problem in the control of Fusarium head blight in China. The resistance is caused by point mutations in the *β2-tubulin*gene. So far, five resistant genotypes (F167Y, E198Q, E198L, E198K and F200Y) have been reported in the field. To establish a high-throughput method for rapid detection of all the five mutations simultaneously, an efficient single-nucleotide-polymorphism-based genotyping method was developed based on the Luminex xMAP system. One pair of amplification primers and five allele specific primer extension probes were designed and optimized to specially distinguish the different genotypes within one single reaction. This method has good extensibility and can be combined with previous reported probes to form a highly integrated tool for species, trichothecene chemotype and MBC resistance detection. Using this method, carbendazim resistant FGSC isolates from Jiangsu, Anhui and Sichuan Province in China were identified. High and moderate frequencies of resistance were observed in Jiangsu and Anhui Province, respectively. Carbendazim resistance in *F. asiaticum* is only observed in the 3ADON genotype. Overall, our method proved to be useful for early detection of MBC resistance in the field and the result aids in the choice of fungicide type.

## Introduction

Increased resistance to the most commonly used fungicide, carbendazim, is escalating Fusarium head blight problems in China. The development of adequate management strategies depends on rapid monitoring of resistance development within present populations. Fusarium head blight (FHB) or head scab is a devastating disease of wheat (*Triticum aestivum* L) and barley (*Hordeum vulgare* L) in China (Zhang et al., 2012; Zhang et al., 2010) and many other regions of the world (Suga et al., 2008; Talas, Parzies & Miedaner, 2011). FHB not only causes quantitative yield losses, but may also reduce grain quality due to contamination with mycotoxins produced by the *Fusarium* pathogens, which pose a significant risk to food safety and animal health (Bai & Shaner, 1996). In recent years, due to the changes in climatic conditions and in agricultural practices, FHB outbreaks have occurred more frequently in China, leading to a significant yield loss in more than 5 million ha (approximately 20%) of wheat grown each year from 2008 to 2015.

Tillage, resistant wheat cultivars and fungicide regimes are regarded as the most important variables for this disease and mycotoxin contamination which can be controlled by growers (Beyer et al., 2006). Tillage is used to remove the crop debris colonized by *Fusarium* species from the surface of the soil. Burning debris was forbidden in China because of the environmental pollution. Currently, the most effective tillage method is inverting the soil and thereby burying crop residues from the previous harvest. This method has been applied in many areas in China. However, in agricultural practice, the ploughing depth is often insufficient due to lack of machinery and their costs, and thereby there are still many crop residues remaining on the surface of the soil. In addition to this, non-tillage seeding is also popular in some areas of China. These facts combined ensure a large amount of initial inoculum for FHB outbreak. Breeding resistant wheat varieties is another important way for FHB control. Although quantitative trait loci (QTLs) of the type I and II resistance were identified (Van Ginkel et al., 1996), no durable, fully FHB-resistant wheat cultivars exist at present, therefore their control relies on the use of commercial cultivars with partial resistance (Mesterhazy et al., 2005). In China, some commercial varieties with moderate resistance to FHB have been widely used in the middle and lower reaches of the Yangtze River Region which is the most severe epidemic area of FHB. However, almost all varieties were highly susceptible to FHB in the Huanghuaihai Region in northern China where roughly 60% of the nation’s output of wheat is produced. Hence, application of synthetic fungicides is the principal method for controlling FHB.

Methyl benzimidazole carbamate (MBC), particularly carbendazim and thiophanate-methyl, have been extensively used to control FHB in China during the past four decades. In addition to this, MBC was also widely used to control many diseases in vegetables and fruits, such as *Botrytis cinerea*. However, wheat is usually planted on large acreage in the main wheat producing areas in China, therefore we think it is unlikely that the application of MBC for other plant diseases influences the FHB pathogens.

The resistance of *Fusarium graminearum* species complex (FGSC) was continuously monitored from 1985 onwards (Ye & Zhou, 1985). MBC-resistant isolates of the FGSC were detected for the first time in 1992 in the Zhejiang Province, while subsequently resistant populations were observed in neighboring provinces in the lower reaches of the Yangtze River including Jiangsu, Shanghai and Anhui, resulting in a decline in the efficiency of control of FHB by MBC (Chen et al., 2007; Liu et al., 2010). Rotational application of several fungicides is an effective way for management of resistant populations and to extend the lifespan of the fungicides. The incidence of resistance is the key information for designing effective fungicide application strategies.

Looking at crop rotations, rice-wheat and maize-wheat rotation within one year are the leading farming systems in Southern and Northern China, respectively. *Fusarium* perithecia on rice and maize debris can be sampled in early April to monitor for fungicide resistance. Infection of wheat heads is expected about three weeks later. Within this short period, strain sampling, isolation and fungicide sensitivity assessment should be finished and the appropriate fungicides should be suggested to farmers. Therefore, high-throughput molecular detection methods are needed to improve detection efficiency and test more isolates, which will be helpful for effective control of FHB.

For conventional detection, pathogens are inoculated in media supplemented with fungicide. This procedure is time consuming and the result is easily influenced by culture conditions and the quality of the chemicals, requiring repeated validation. Therefore, it is difficult to test large populations in a short time. Along with the elucidation of the resistance mechanism of some fungicides, molecular techniques are used more and more for rapid detection of fungicide resistance. Point mutations in the *β*-*tubulin* gene confer resistance against MBC fungicides in various phytopathogenic fungi (Albertini, Gredt & Leroux, 1999; Koenraadt, Somerville & Jones, 1992; Ma & Michailides, 2005; Pierre et al., 2002). In FGSC, there are two homologous *β*-tubulin genes (*Tub1* and *Tub2*) in the genome. Recent study showed the resistance of FGSC to MBC is primarily due to mutations in *Tub2* (FGSG06611) rather than *Tub1* (FGSG09530) (Chen et al., 2009). To date, at least five different point mutations of *Tub2* gene conferring MBC resistance have been detected in the field isolates. Based on the SNPs, several molecular techniques have been developed for rapid detection of MBC-resistant isolates. Liu et al. (2010) developed three pairs of allele-specific PCR (ASPCR) primers to detect the resistant isolates harboring F167Y, E198Q and F200Y mutations respectively. PIRA-PCR and cycleave PCR approaches were developed for the specific detection of the F167Y genotype (Hou et al., 2011; Luo et al., 2009). Liu et al. (2014) developed real-time PCR assays for quantitative detection of the frequency of five alleles (F167Y, F200Y, E198Q, E198L and E198K). Recently, a simple on-site loop-mediated isothermal amplification (LAMP) method was also used for detecting resistant isolates with F167Y and F200Y mutation (Duan et al., 2014; Duan et al., 2016). All the methods above could detect carbendazim resistance much more rapidly than the classical mycelial growth inhibition assay. However, all of these methods, require a separate reaction for each target genotype per sample. Thus, the number of assays are equal to the product of the number of genotypes that can be detected and the number of samples. This results in elevated workload. Therefore, a high-throughput method is needed for simultaneous detection of any of the known MBC resistant genotypes in FGSC.

Luminex xMAP is a microsphere-based multiplexing system where microspheres are internally dyed with various proportions of red and infrared fluorescent dyes, producing different spectral addresses detected by two lasers. User-designed specific probes can then be bound to these microspheres and tested in a 96-well format using the biotinylated PCR or extension products with hybridization reactions quantified by the fluorescence of the reporter molecule streptavidin-R-phycoerythrin. Based on this high-throughput system, several nucleic acid assays have been developed. Direct DNA Hybridization Sequence Detection (DHSD) is the most common assay that can be used for specific sequence detection. It has been previously employed for genotyping a wide range of microorganisms, such as *Aspergillus* species (Etienne, Kano & Balajee, 2009), *Candida* species (Das et al., 2006) and others. Ishii et al. (2008) applied this DHSD method for detection of fungicide resistance for the first time. They succeed in detecting strains resistant *Magnaporthe oryzae* to inhibitors of scytalone dehydratase in melanin biosynthesis (MBI-D). However, using the direct probe hybridization to detect SNPs usually requires extensive optimization, and background hybridization is often high. Multilocus Genotyping Assay (MLGT) is a flexible, simple and robust chemistry that is quite suitable for detecting SNPs and other sequence variations. MLGT requires Allele Specific Primer Extension (ASPE) probes that are complementary to the target sequence. When the 3^′^-end base of the probe is complementary, the polymerase can use it to synthesize new DNA containing biotin labeled nucleotides, but a primer cannot promote this extension if its 3^′^-end base is non-complementary. The flexibility of the assay has allowed its use for genotyping a number of different targets in one reaction. Ducey et al. (2007) developed 60 ASPE probes in nine genome regions for subtyping lineage I isolates of *Listeria monocytogenes*. MLGT were also used for species and trichothecene chemotypes identification of FGSC with at least 48 ASPE probes (Sarver et al., 2011; Ward et al., 2008).

We developed a fast SNPs-based genotyping assay for the detection of all the five known MBC resistant genotypes of FGSC, which can be used complementary to the time consuming antifungal resistance tests based on plating and cultivation. We demonstrate that it can be combined with the previously reported method to detect *Fusarium* species and chemotypes simultaneously and that the assay is efficient to provide critical information for fungicide application in the field. The new method will be used for early warning of resistance risks of FHB pathogens to MBC fungicides in fields. Combined with monitoring resistance frequency to other fungicides, suggestions for the rotation of different fungicide groups can be made and will be helpful for a sustainable protection against the fungus over time.

## Material and Methods

### *Fusarium* isolates and vectors

*F. asiaticum* isolate js205, which is highly resistant to MBC because of a point mutation at codon 167 (Phe to Tyr, F167Y) in the *Tub2* gene, *F. asiaticum* isolate js161, which is highly resistant to MBC because of a point mutation at codon 200 (Phe to Tyr, F200Y) in the *Tub2* gene, *F. asiaticum* isolate js801, which is moderately resistant to MBC because of a point mutation at codon 198 (Glu to Gln, E198Q) in the *Tub2* gene and *F. asiaticum* isolate js166, which is sensitive to MBC, were used as reference in this study (Table 1). We introduced the E198L and E198K mutation respectively to the wild type *Tub2* gene artificially to make recombinant plasmids for described mutant types that we didn’t have in this study. The plasmids were used for validation of probes targeting these mutations. For E198L, parts of *Tub2* gene were amplified by primer pairs Tub2GF/198LR and 198LF/Tub2GR, respectively, and the genomic DNA of sensitive isolate js166 was used as template. The two fragments were joined by double-jointed PCR using primers Tub2GF/Tub2GR and cloned into cloning vector pMD19T-Simple (TaKaRa) to generate pMD-198L. Using primers 198KF/198KR instead of 198LF/198LR, the same procedure was used to construct the vector pMD-198K containing the E198K mutation. Genomic DNA of *Fusarium* isolates were extracted using the SP Fungal DNA Kit (OMEGA) according to the manufacturer’s instructions.

**Table 1:** FGSC isolates used in the validation of MLGT.

Isolates	Origin	Genotype description^a^	EC_50_ (µg mL^−1^)	Resistance level^b^
js166	Jiangyan, Jiangsu Province	Wild type (KX061865)	0.60	S
js205	Baoying, Jiangsu Province	F167Y (KX061866)	10.13	HR
js161	Jiangyan, Jiangsu Province	F200Y (KX061867)	12.37	HR
js801	Gaoyou, Jiangsu Province	E198Q (KX061868)	3.54	MR

**Notes.**

a The number in the bracket is the GenBank accession number of partial *Tub2* gene sequence.

b S, MR, and HR indicate that the isolates are sensitive, moderately resistant, and highly resistant to carbendazim, respectively.

In 2014, a total of 156 single-spore *Fusarium* isolates were collected from infected wheat heads in six sampling sites distributed in three provinces—Jiangsu, Anhui and Sichuan. Carbendazim has been used continually for more than 30 years in Jiangsu Province. It has been used since 2008 in Anhui Province and has seldom been used in Sichuan Province.

### Mycelial growth inhibition assay for MBC resistance determination

Technical-grade carbendazim (98% active ingredient (AI); Shanghai Shennong Pesticide Co., Ltd, Shanghai, China) was dissolved in 0.1Mhydrochloric acid (HCl) toprovide stock solutions containing 10 mg ml^−1^. The fungicide was added to potato dextrose agar (PDA) after autoclaving at different concentrations. Single 5-mm mycelial plugs were taken from the edge of a 2-days-old colony of each isolate and placed on each concentration of fungicide amended PDA. Four replicated plates were used for each isolate. Petri plates were incubated at 25 °C for 3 days in the dark and then radial growth of each isolate was measured. The sensitivity of tested strains were determined according to the reported discriminatory concentration of 1.4 µg ml^−1^ of carbendazim (Zhou & Wang, 2001). Strains with a minimum inhibitory concentration (MIC) > 1.4 µg ml^−1^ were regarded as resistant isolates and the ones with lower MIC values as sensitive isolates. The concentrations of 0, 3.125, 6.25, 12.5, 25, 50, and 100 µg ml^−1^ were used to test the carbendazim sensitivity and calculate the EC_50_ of the resistant reference isolates –js205, js161 and js801 and the concentrations of 0, 0.2, 0.4, 0.6, 0.8, 1.0, and 1.2 µg ml^−1^ were similarly used for the sensitive isolate js166.

### Multiplex amplification of template for MLGT assay

Tub2F/Tub2R were used to amplify a part of the *Tub2* gene containing the SNPs associated with MBC resistance (Table 2). In order to detect species, chemotypes and MBC resistant genotypes simultaneously, we combined Tub2F/Tub2R and six previously developed primer pairs (Ward et al., 2008) for a multiplex amplification. Amplifications were performed in 20 µl volumes with PrimeSTAR HS Premix (Takara), and approximately 100 ng of genomic DNA. PCR conditions consisted of an initial denaturation of 90 s at 94 °C, followed by 40 cycles of 30 s at 94 °C, 30 s at 50 °C, and 1 min at 68 °C. PCR products were treated with ExoSAP-IT (USB) to remove primers and unincorporated dNTPs and served as template for allele-specific primer extension reactions.

**Table 2:** Primers used in this study.

Primer	Sequence 5^′^–3^′^
Tub2GF	ATGCGTGAGATTGTCCACGTCC
Tub2GR	TCAACCCTCGTACTCCTCGGGC
198KF	TCTGACAAGACCTTCTGTATCGATA
198KR	ATACAGAAGGTCTTGTCAGAGTTCTCG
198LF	ACTCTGACCTGACCTTCTGTATCGATAACGAG
198LR	CGTTATCGATACAGAAGGTCAGGTCAGAGTTCT
Tub2F	GCTGACGCACTCTCTCGGCG
Tub2R	CGGCCATGACGGTGGAAATC

### Probe design and extension reactions for the MLGT assay

Five different point mutations of *Tub2* gene conferring MBC resistance to *F. graminearum* in the field have been reported, including codons 167 (TTT to TAT, F167Y), 198 (GAG to CTG, E198L; GAG to CAG, E198K; GAG to AAG, E198Q) and 200 (TTC to TAC, F200Y). Based on this, oligonucleotide probes with 3^′^-end nucleotide specific to individual SNPs were designed (Table 3). Each of the probes was synthesized with a unique 24 bp sequence tag on the 5^′^-end that was specific to an anti-tag sequence of individual set of Magplex-xTAG microspheres (Luminex Corporation) (Table 3). Extension reactions were performed in standard 20 µl reaction mixtures according to manufacturer specifications and included 1.25 mM MgCl_2_, 5 µM biotin-dCTP, 5 µM dATP/dGTP/dTTP, 25 nM of each probes, 0.75 U Platinum Geno-TYPE *Tsp* DNA polymerase (Invitrogen). Five microliters of purified multiplex PCR products was added as the template for extension reactions, which were performed with an initial denaturation of 2 min at 96 °C, followed by 40 cycles of 30 s at 94 °C, 1 min at 55 °C, and 2 min at 74 °C.

**Table 3:** ASPE probes and probe performance data.

Probe^a^	Target	Sequence^b^	MFI^c^		ID^d^
			Positive	Negative	
167F (20)	F167Y	CTTTCTCATACTTTCAACTAATTTtcgcatgatggccaccta	1,929–2,725	35–98	19.6
198F (21)	E198Q E198L	TCAAACTCTCAATTCTTACTTAATcagctcgtcgagaactctgac	1,832–2,433	45–153	12.0
198R1 (22)	E198L	CAAACAAACATTCAAATATCAATcctcgttatcgatacagaaggtca	1,683–1,999	36–205	9.8
198R2 (25)	E198K	CTTTCTTAATACATTACAACATACcctcgttatcgatacagaaggtctt	2,861–3,493	16–108	26.7
200R (26)	F200Y	TACATTCAACACTCTTAAATCAAAcagagcctcgttatcgatacagt	2,325–3,378	28–305	11.1

**Notes.**

a The number in the bracket represent the MagPlex-TAG™ microsphere sets.

b The 50 sequence tag portions of extension probes are capitalized.

c The range of MFI (Median Fluorescence Intensity) values are reported for isolates representing targeted and non-targeted resistant genotypes from the reference isolates and *Fusarium* population in Jiangsu, Anhui and Sichuan Province.

d Index of discrimination (ID) value, determined as minimum positive MFI/maximum negative MFI.

### Hybridization and detection for the MLGT assay

Biotinylated extension products were hybridized with a mix of microsphere sets specific to each of the sequence tags appended to the 5^′^end of the extension probes. Hybridization reactions were performed in 50 µl volumes with 1 × TM buffer (Sigma), 10 µl of extension product, and 1,250 microspheres from each set. The samples were incubated for 90 s at 96 °C, followed by 45 min at 37 °C. Microspheres were pelleted twice by centrifugation and resuspended in 75 µl TM buffer containing 2 µg/ml streptavidin-R-phycoerythrin. Samples were incubated for 15 min at 37 °C prior to detecting the microsphere complexes with a Luminex 200 flow cytometer. The median fluorescence intensity (MFI) from biotinylated extension products attached to 100 microspheres was measured for each probe. An index of discrimination (ID) was calculated as the ratio of the lowest positive MFI to the highest negative MFI value for each probe. Probes with a ratio of less than 3.0 were redesigned. Five reference strains and two recombinant vectors are used to validate the probes. Each isolate was genotyped via five independent runs of the MLGT assay.

### Application of MLGT on monitoring MBC-resistance of *Fusarium* population in three provinces

To directly demonstrate the application of MLGT on resistance monitoring of *Fusarium* isolates to carbendazim in agricultural production, 156 *Fusarium* isolates collected from diseased wheat heads in three provinces along the Yangtze River in 2014 were tested with this method. At the same time, MIC method with the discriminatory concentration of 1.4 µg ml^−1^ of carbendazim was performed on the same population to validate the MLGT result. To test the compatibility of the new probes and previously developed probes by Ward et al. (2008), seventeen species- and trichothecene-chemotype- specific probes were selected and combined with the five MBC resistance probes to perform the MLGT assay simultaneously. The selected probes are specific to B-FHB clade, FGSC, *F*. *graminearum*, *F. asiaticum*, *F. meridionale*, *F. boothii*, 3ADON, 15ADON and NIV respectively, which are the prevalent FGSC species and chemotypes in China. The selected probes and multiplex PCR primers are summarized in Table S1.

## Results

### Design and validation of the SNP-based genotyping assay

Based on mycelial growth inhibition assay, isolates js205 and js161 were regarded as highly resistant (HR) with EC_50_ higher than 10 µg ml^−1^, isolate js801 showed moderately resistance with an EC_50_ of 3.53 µg ml^−1^, while strain js166 was regarded sensitive with an EC_50_ value of 0.60 µg ml^−1^. Sequencing analysis showed that point mutations F167Y, E198Q and F200Y were present in the *Tub2* gene of these isolates, respectively (Table 1). In addition to the three common mutations, E198L and E198K have also been reported to occur at very low frequencies in the field (Liu et al., 2014). Because we did not have strains with these two genotypes, we introduced the two mutations into the *Tub2* gene artificially and constructed vectors pMD-198L and pMD-198K. These strains and vectors were used for validation of MLGT assay.

Based on the SNPs in *Tub2* gene, five ASPE probes were designed to identify all the MBC resistant genotypes (Table 3). 167F and 200R were specific to F167Y and F200Y genotypes, respectively. The other probes were used to identify the three mutations in 198 codon. 198R2 was the specific probe for E198K. There were two SNPs in E198L, including the G to C mutation which also caused E198Q. Therefore, these two genotypes should be determined by both 198F and 198R1 probes. Positive 198F and negative 198R1 represent E198Q genotype, while positive of both probes indicate E198L genotype. As shown in Table 4, all five probes were negative for the MBC sensitive isolate js166, positive MFI of 167F (2,261 ± 79) and 200R (2,576 ± 251) were observed for highly resistant isolates js205 and js161 respectively. Moderately resistant isolate js801 showed positive 198F (2,266 ± 167) and negative 198R1 (72 ± 19), which indicated an E198Q genotype. Using pMD-198L as template, 198F (1987 ± 69) and 198R1 (1,786 ± 103) were both positive, as expected. 198R2 (3,216 ± 277) was positive for pMD-198K. In order to test whether the presence of genomic DNA influenced the efficiency of 198R1 and 198R2, the plasmids pMD-198L or pMD-198K were mixed with the genomic DNA of sensitive isolate js166. Similar results were observed indicating that 198R1 and 198R2 were suitable for identifying E198L and E198K in practice. For all the five probes, the lowest index of discrimination (ID) was 10.2 (198R1). This indicated that the probes are robust in distinguishing the resistant mutations and the wild type.

**Table 4:** Validation of the MLGT method by reference strains.^a^

Probes					
Strains	167F	198F	198R1	198R2	200R
js166	57 ± 22	71 ± 7	76 ± 23	48 ± 28	124 ± 21
js205	2,261 ± 79	102 ± 19	54 ± 13	65 ± 33	79 ± 12
js161	65 ± 19	82 ± 32	134 ± 31	75 ± 19	2,576 ± 251
js801	62 ± 15	2,266 ± 167	72 ± 19	62 ± 21	57 ± 22
pMD-198L	68 ± 25	1,987 ± 69	1,786 ± 103	72 ± 31	89 ± 34
pMD-198K	39 ± 21	85 ± 27	88 ± 40	3,216 ± 277	117 ± 18
js166+pMD-198L	55 ± 18	2,376 ± 37	1,913 ± 86	54 ± 16	159 ± 46
js166+pMD-198K	73 ± 20	59 ± 12	101 ± 41	2,987 ± 126	69 ± 23

**Notes.**

a The number in the table are average MFI (median fluorescence intensity) values.

**Table 5:** Summary of species, chemotype and MBC resistance frequency of FGSC populations from Jiangsu, Anhui and Sichuan Province.

Provinces	Sampling sites	Number of isolates	*F. graminearum*	*F. meridionale*	*F. asiaticum*		Carbendazim resistance^b^		CarR isolates carrying the point mutation^c^		
			15ADON	NIV	NIV	3ADON	Car^*R*^	Resistance frequency^d^	F167Y	E198Q	F200Y
Jiangsu	Gaoyou	27	2	0	0	25 (7)^a^	7	20.34 ± 10.48%^a^	6	1	0
	Yangzhou	32	1	0	2	29 (5)	5		4	0	1
Anhui	Fengtai	31	5	0	1	25 (2)	2	9.09 ± 7.61%^b^	2	0	0
	Xuanzhou	35	0	0	0	35 (4)	4		3	1	0
Sichuan	Mianyang	31	6	2	20	3	0	0.00 ± 7.85%^b,c^	0	0	0
	total	156	14	2	23	117 (18)	18	11.54 ± 5.23 %	15	2	1

**Notes.**

a The number in the bracket is the number of MBC-resistant isolates within this species and chemotype.

b Carbendazim resistance were tested at the discriminatory concentration of 1.4 µg mL^−1^ on PDA plates.

c Different resistant genotypes of *Tub2* gene were determined by MLGT.

d Frequencies with a letter in common do not differ statistically according to Chi^2−^tests (*p* < 0.05). The confidence intervals were calculated using the adjusted Wald method.

### Application of MLGT on monitoring MBC-resistance of *Fusarium* population in three provinces

To demonstrate the application of MLGT in practice, MLGT was performed on a *Fusarium* population collected from five sampling sites in three provinces along the Yangtze River. Out of the 156 *Fusarium* isolates tested, eighteen (11.5%) strains were carbendazim-resistant. Most resistant isolates (*N* = 15, 83.3%) had the point mutation F167Y, two (11.1%) carried the point mutation E198Q and the remaining one (5.6%) harbored the point mutation F200Y. No strains carrying the E198L or E198K mutations were found (Table 5). Based on the result of this population, we summarized the MFI range of the five probes and calculate the index of discrimination. The ID values of the probes ranged from 9.8 to 26.7 (Table 3), which means the MFI values for isolates with a positive genotype were at least 9.8 times higher than the MFI values for isolates with a negative genotype. Because of the significant MFI difference, positive and negative genotypes can be distinguished clearly.

To validate the result of the MLGT, sensitivity of this *Fusarium* population to carbendazim was also evaluated by MIC method. All the 18 isolates with point mutations F167Y, E198Q or E200Y identified by MLGT were resistant to carbendazim (MIC > 1.4 µg ml^−1^) (Table 5), while all other isolates proved to be sensitive to the discriminatory concentration of carbendazim (MIC: 1.4 µg ml^−1^). The consistent results from these two approaches revealed that the MLGT method is reliable and can be used for application in the field.

The frequency of carbendazim resistance is different in the different regions and the three provinces; the Jiangsu population showed the highest incidence of resistance (15.60–25.90%), the frequency in Anhui is lower (6.25–11.40%), while no resistant isolates were found in Sichuan. Chi^2^-tests confirmed the differences even with relative low samples sizes Jiangsu and Sichuan (*p* = 0.005) and Jiangsu and Anhui (*p* = 0.003) differ significantly, while Anhui and Sichuan (*p* = 0.078) are not significantly different. Looking at the five regions sample separately, there is a clear increase in resistant colonies from North to South (Table 5).

We combined seventeen previously developed probes and the new MBC resistant probes in a single reaction to detect species, chemotypes and carbendazim resistance simultaneously. All probes worked well and did not influence the efficiency of each other. Based on the significant MFI difference, all SNPs can be clearly distinguished (Table S2). All isolates tested belong to FGSC and three species were identified. *F. asiaticum* was the predominant species (*N* = 140, 89.7%) in all sampling sites. *F. graminearum* was also found in most sampling sites (4/5) with a frequency of 9.0%. Two *F. meridionale* isolates were identified in Sichuan Province. The chemotype of all *F. graminearum* isolates were of the 15ADON chemotype and *F. meridionale* isolates were of the NIV type. Most *F. asiaticum* were 3ADON type (*N* = 117, 83.6%) and others were NIV producers (*N* = 23, 16.4%). NIV and 3ADON producers showed a strong association with their geographical origin. 3ADON producers (91.2%) dominate Jiangsu and Anhui Province, while 71.0% of the isolates produce NIV in Sichuan Province. All carbendazim resistant isolates were *F. asiaticum* with 3ADON type (Table 5). The frequency of carbendazim resistant colonies in 3ADON type *F. asiaticum* strains (18/117) is significantly higher than in NIV-producing *F. asiaticum* (0/23) (Chi^2^test; *p* = 0.041), whereas for NIV* F. meridionale* and 15ADON-*F. graminearum* the samples were too small to compare with (Table 5).

## Discussion

Fusarium head blight is the most important wheat disease in the areas along the Yangtze River in China, leading to a severe mycotoxin contamination of wheat products. Control of this disease is largely dependent on the use of synthetic fungicides, because few cultivars with effective resistance are available. However, the efficacy of fungicides in the field is limited by development of resistance. Treatment options depend on the presence and frequency of resistant isolates. The time window from one crop to the next in which strategy should be decided is approximately three weeks. Our luminex-based technique allows the high throughput analysis needed for adequate screening for the presence of resistant strains.

Carbendazim is the most widely used fungicide to control FHB in China,where it was first used in 1972 in Jiangsu Province. The resistance of FGSC was continuously monitored from 1985 onwards (Ye & Zhou, 1985). The first MBC-resistant strain was isolated in 1992 in Zhejiang Province and two years later resistant isolates were found in the neighboring province Jiangsu. Zhang et al. (2009a) and Zhang et al. (2009b) monitored the frequency of resistant isolates from 1985–2008 in Jiangsu, where they found a close relationship between the frequency of MBC-resistant isolates in the field and FHB severity. During sever FHB outbreaks, farmers applied more MBC, increasing the selective pressure for resistance, which lead to increased resistance frequencies in the following years. However, the years following the sever outbreak had lower disease index and many farmers did not feel the need to apply MBC. The decrease in the application of MBC removed the selective advantage of resistant isolates, and the frequency of resistance decreased in the region within five years to the resistance frequency observed before the outbreak (Zhang et al., 2009a). This indicated that rotation with other fungicides could be effective to manage MBC resistance. From 2008, FHB outbreaks are more frequent in China and a significantly high frequency of carbendazim resistant isolates was found in the southern part (Duan et al., 2014; Liu et al., 2014). It is reported that when resistant isolates account for 5 or 10% in a *Fusarium* population, efficacies of carbendazim are reduced by 10 or 37.5% respectively. Carbendazim resistance can also lead to an increase in trichothecene production (Zhang et al., 2009b). Therefore, monitoring carbendazim resistance frequency in the FGSC population is important for effectively controlling FHB and mycotoxin contaminations.

In this study, we succeeded in applying a SNP-based genotyping assay for carbendazim resistance detection of FGSC. Adding the five newly developed ASPE probes and the primer pair specific to the *Tub2* gene resulted in the development of a high-throughput method that can detect any of the known MBC resistant genotypes in FGSC populations. All of the five ASPE probes showed high ID value (9.8–26.7), indicating the positive and negative MFI can be distinguished clearly. So far, five resistant genotypes of FGSC have been characterized. Based on the previous reports (more than 10,000 isolates tested) in China (Duan et al., 2014; Liu et al., 2010; Liu et al., 2014), F167Y was predominant (>80%), followed by E198Q and F200Y. These three genotypes covered 99.99% tested resistant isolates. The ratio of E198L and E198K was extremely low. This indicate F167Y, E198Q and F200Y have advantages in the MBC selection. Therefore, the five resistant genotypes detected in this method can represent the MBC resistant population and give a comprehensive result. Even if other unknown resistant genotypes exist, their low frequency, like that of E198L and E198K cannot influence the resistance frequency. xMAP technology has been used for MBI-D resistance detection of *Magnaporthe oryzae* by a direct probe hybridization method (Ishii et al., 2008). However, using the direct probe hybridization to detect one SNP usually requires strict control of the reactive conditions and extensive optimizations. The background hybridization of this method is often high. This limits the number of targets involved in one reaction significantly. Therefore, this study is the first to apply SNP-based detection of multiple SNPs conferring fungicide resistance inside an MLGT system. MLGT is an open platform, usually new probes can be added into a former system with little difficulty. Ward et al. (2008) developed 41 probes for species and chemotype identification of FGSC and closely related B-FHB species. Along with the consistent discovery of new species, the number of probes has been extend to 48 (Aoki et al., 2015). In this study, we also succeed to combine the new MBC resistant probes and the previously reported probes in a single reaction to detect the carbendazim resistance, species and chemotype simultaneously. This means when new SNPs associated with resistance to carbendazim or other fungicides are characterized, the probes panel can be expanded to detect the new genotypes as well. Using this integrated approach, more than 50 targets including fungicide resistant genotypes of at least 384 samples can be performed within a single day, which is much faster and cheaper than mycelial growth inhibition assay (4–5 days) and other separate molecular detection techniques. Furthermore, this method eliminates a large amount of parallel work. Besides resistance, this method can also provide information on their resistant genotypes. Based on this, dynamic of the five resistant genotypes in different regions can be monitored, this is helpful for understanding the adaptive spread of these point mutations. It is reported that different point mutations showed different resistance level, we also validated in this study. Therefore, we can also get the ratio of isolates with different resistance level from the MLGT result. This is important information, because this ratio can also influence the overall resistance level of the population.

In China, four species within FGSC have been identified on wheat (Zhang et al., 2012) and on maize (Zhang et al., 2016). In this study, seventeen probes specific to these four species and three trichothecene chemotypes were used to test the *Fusarium* population collected along the Yangtze River together with the five MBC resistant probes. In agreement with previous reports, the frequency of resistance in the FHB populations was correlated with with the frequency of carbendazim usage in the different provinces (Duan et al., 2014; Liu et al., 2014). The highest frequency of resistance was observed in Jiangsu Province where carbendazim had been used continually for more than 40 years. The FHB population in Anhui Province, where carbendazim has been applied since 2008, showed a lower frequency of resistance. According to these results, tank mixtures or other fungicides with different modes of action such as tebuconazole and triadimefon are suggested to replace carbendazim in Jiangsu and Anhui Province. Carbendazim had been seldom used in Sichuan Province, and no resistant isolates were found there. There have been no MBC resistant strains reported in Northern China so far. Therefore, monitoring MBC resistance is more important in these areas.

Several studies report that F167Y was the most common resistant genotype (Chen et al., 2009; Liu et al., 2010). In this study, we also found F167Y to be the predominant resistant genotype in both Jiangsu and Anhui Province followed by E198Q and F200Y. So far, only two isolates harboring E198L and one isolate with E198K were identified in the field (Chen et al., 2009; Liu et al., 2014). We did not find these genotypes in this study probably due to their extremely low frequency and the small size of the population tested. The mycelial growth inhibition assay showed consistent results, indicating that the MLGT method is reliable. In addition to carbendazim resistance, species and chemotypes of the *Fusarium* populations were determined simultaneously. In agreement with previous reports (Yang et al., 2008; Zhang et al., 2012), *F. asiaticum* dominated the regions along the Yangtze River and *F*. *graminearum* was found in most sampling sites at low frequency. *F. meridionale* was only found in the mountain regions of Sichuan Province. 3ADON and NIV producers dominate the lower (Jiangsu and Anhui) and upper reaches (Sichuan) of the Yangtze River, respectively. In this study, we observed carbendazim resistance exclusively associated with *F. asiaticum* with 3ADON genotype (Table S2). This may be due to the long history and high dosage of carbendazim usage in the regions along the lower reaches of the Yangtze River, where 3ADON producing *F. asiaticum* is predominant. We previously found that *F. asiaticum* with 3ADON genotype showed several advantages over the NIV population, including growth rate and pathogenicity. The larger population size resulting from these characteristics made it more probable that this population would develop a resistant genotype to MBC. To validate whether 3ADON *F. asiaticum* is the only genotype that has resistant members, an extended sampling of the *Fusarium* population of China should be undertaken. It is also reported that carbendazim resistance can increase DON production and biomass accumulation in wheat heads significantly (Zhang et al., 2009b). This would also imply that resistant populations have a higher fitness in the environment and may be favored by natural selection.

In conclusion, a multilocus genotyping method was established and demonstrated to be high-throughput and more accurate and practical for detection of the resistant FHB pathogens to MBC fungicides than previous methods. It is an open platform and can be integrated with the species and chemotype detection approach and other new probes that may be developed in the further. Therefore, it will be potentially useful for monitoring MBC fungicides resistance in FGSC population and design practical disease management strategies in the future.

##  Supplemental Information

10.7717/peerj.2609/supp-1 Table S1 Previously developed probes (Ward et al., 2008) used in this study

10.7717/peerj.2609/supp-2 Table S2 MFI of 17 probes on 156 Fusarium isolates form Jiangsu, Anhui and Sichuan Province

10.7717/peerj.2609/supp-3 Supplemental Information 1 The second run of raw data exported from Luminex 200 applied forcarbendazim resistance, species and chemotype detection of 156 *Fusarium* isolates

10.7717/peerj.2609/supp-4 Supplemental Information 2 Raw data generated by Luminex

## Additional Information and Declarations

### DNA Deposition

The following information was supplied regarding the deposition of DNA sequences: GenBank: KX061865, KX061866, KX061867, KX061868.

### Data Availability

The following information was supplied regarding data availability: The raw data has been supplied as Supplemental Dataset.
